# The experience of informal caregiving within Saudi society: expressed needs and expectations

**DOI:** 10.1186/s42506-023-00130-y

**Published:** 2023-02-15

**Authors:** Soha Almehmadi, Abeer M. Alrashed

**Affiliations:** College of Business Administration, King Saud University, Riyadh, Saudi Arabia

**Keywords:** Informal caregivers, Elderly, Long-term care, Individuals with disability, Saudi society

## Abstract

**Background:**

Even though informal caregivers have always been a major element within any society, their contribution to the healthcare system has only recently been recognized. Accordingly, the sustainability of their informal social role is becoming a major concern to policymakers. In Saudi Arabia, recognition of informal caregiving is still limited. This study was carried out to investigate the experience of caregiving in informal settings through identifying the needs of the caregivers along with their expectations of the formal system.

**Methods:**

A cross-sectional study was conducted to measure the experience of Saudi informal caregivers who were caring for at least one individual with a dependency resulting from either disability, aging, or both. A self-administered questionnaire was designed specifically for this study with 88% reliability. A convenient sample of 300 caregivers was invited electronically through 14 websites supporting families with dependent individuals.

**Results:**

Of the eligible participants (*n* = 271), about two-thirds were caring for one elderly person or more, while one-third were caring for individual(s) with disabilities. The two groups did not differ in their needs; their greatest perceived needs were necessary equipment for care recipients, free time to socialize, alternative care setting, and proper income. Overall, the needs on the system level were the highest, followed by the needs on the financial level, then on the social level. On the other hand, the caregivers of individuals with disabilities had greater expectations of formal support than caregivers of the elderly. The greatest expectation among the participants was facilitating care recipients’ mobility within their communities. Overall, the expectations of information support were the highest followed by the expectations of financial support and then material support.

**Conclusion:**

The participants expressed great expectations of formal support along with some unmet needs. Further research is recommended to emphasize the role of primary caregivers along with the nature of the assistance received throughout the caregiving process. The needs of individuals with dependencies along with those of the caregivers must be considered in the planning process of healthcare services. Finally, the expectations of informal caregivers should lead the priorities of the development decisions of long-term care services.

## Introduction

Long-term care services include a broad range of health, personal care, and supportive services that meet the needs of frail older people and other adults whose capacity for self-care is limited because of a chronic illness; injury; physical, cognitive, or mental disability; or other health-related conditions [1]. The process of long-term caregiving arises out of a relationship between a caregiver and a recipient with a certain level of dependency [2]. There are two kinds of caregiving: First is the formal care which is provided by paid regulated providers within a formal system. Second is the informal care which is provided by caregivers outside the boundaries of any formal system [3, 4].

Formal caregivers are clinicians and trained individuals who receive compensation to provide intermittent or continuous healthcare services [3]. On the other hand, informal caregivers are individuals, often family members, who provide care, typically unpaid, to someone with whom they have a personal relationship [5]. In this study, the term caregiver refers to informal caregivers unless otherwise specified.

Care recipients are individuals who depend on others to perform their daily activities due to incapacity resulting from aging or impairment, temporary or permanent. The most common forms of care received are assistance with activities of daily living (ADLs) or instrumental activities of daily living (IADLs) and care coordination, which involves figuring out what kind of care is needed, where to find care, and how to arrange for care [3].

Many of the very elderly lose their ability to live independently because of limited mobility, frailty, or other physical or mental health problems. They may require some form of long-term care, which can include home nursing, community care and assisted living, residential care, and long stays in hospitals [2]. In 2015, the World Health Organization (WHO) projected that between the years 2015 and 2050, the number of people aged 60 years and older is expected to double from 900 million (12%) to two billion (22%) [6] whereas its report on disability stated that around 785 million (15.6%) persons aged 15 years and older live with disabilities. Of those, 2.2% are estimated to have very significant difficulties in functioning [7]. In 2021, the WHO estimated that over one billion (15%) persons aged 15 years and older live with disability. Of those, 190 million (3.8%) have significant difficulties in functioning [5].

In developing countries, the number of older people who are no longer able to look after themselves is forecasted to quadruple by 2050 [8]. In Saudi Arabia, as well as globally, life expectancy is on the rise; from the period 1980–1985 to the period 2005–2010, it increased from 64.9 to 74.3 years and is projected to reach 81.8 years during the period 2045–2050 [9]. On the other hand, the number of individuals with disabilities in Saudi Arabia cannot be accurately estimated because the majority of the related surveys have focused on cross-sectional, community-based epidemiology [10]. However, it is estimated that 3.73% of the population have functional disabilities, which limit their independence [8]. Yet, according to a local study, the countrywide data from the demographic survey have indicated that about 0.8% of the total Saudi population have disabilities [9].

Globally, studies were conducted to investigate the support needed by informal caregivers of the elderly [10–15]. Although researchers had investigated caregiving in different societies, their findings were similar to a great extent. Mostly elderly care is provided at homes; some caregivers reported receiving little support while others provided the care with no support at all. Caregivers identified their role of caring to be a positive experience despite experiencing some difficulties during the caring process. Social support was pointed out a lot as an unsatisfied need along with, mainly in developing countries, financial support. Some mechanisms reported to be effective in enhancing the caregiving process were effective communication with the formal system, availability of information on the recipients’ conditions and needs, receiving training on the essentials of caregiving, and respite care [12–17].

Furthermore, prior studies investigated the needs of informal caregivers who provided care for individuals with needs that vary from transitory conditions to long-lasting impairments such as cancer, AIDS, autism, and organ transplant [18–25]. The surveyed caregivers were expecting greater support from the healthcare system especially when caring for individuals with medical conditions that involve some sort of medical intervention. Regardless of the study location, most of the findings reported unmet needs of the informal caregivers such as social support, emotional support, and financial support, in addition to an emphasized need for training courses on the caring process.

However, except for a few studies, the reviewed literature revealed findings representing experiences within systems other than the Saudi health system where most of them had investigated the needs among relatives, rather than caregivers, of individuals who have been cared for by formal caregivers in either a hospital or other formal healthcare settings [26–28].

Informal caregivers are a critical resource to their care recipients and an essential component of the healthcare system worldwide, yet their role and importance to the society have only recently been appreciated. In Saudi Arabia, informal caregivers are still unrecognized as concluded by a local study conducted in 2016 [29]. This study aimed to investigate the experience of caregivers in informal settings within the Saudi society through identifying the caregivers’ s needs along with their expectations of the formal system.

## Methods

### Study design

A cross-sectional design using an online survey was utilized.

### Operational definitions of terms

Informal caregiving refers to providing assistance and personal care that are not under any obligations of a formal structure. Care recipients are those individuals who need support in performing their daily activities due to the natural process of aging or due to impairments, whether permanent or temporary. Activities of daily living (ADLs) that may require a certain degree of dependency are bathing, dressing, toileting, ambulating, managing incontinence, and feeding. The experience of caregiving is measured by defining the needs (must have) and the expectations (could have) of the caregivers.

### Study participants

Caregivers who are caring for at least one individual with a dependency resulting from either disability, aging, or both were invited to participate through a non-random sampling procedure. The invitation along with the questionnaire was distributed electronically through 14 websites of groups and communities supporting families with dependent individuals in the Kingdom of Saudi Arabia. Those groups include the blind association charity in Riyadh, “Roya” blind association charity in Madina, “Moltaga” community for special needs individuals, “Twahodvoice” community to support families having children with autism, human rights Saudi Arabia website, patients’ rights and relations website, autism community support blog, deaf club Riyadh, Jish organization, Akennah association charity for the elderly, aged care association, Alihssan human care charity association, Kenan volunteer group, and National Charity for Home Health Care.

The inclusion criteria were to be a Saudi caregiver, providing care in an informal setting, providing care without any payments related directly to the caregiving, able to read the Arabic language, and residing in Saudi Arabia for at least 1 year prior to data collection. Participation was voluntary and based on research ethics that was approved by the committee of research ethics at King Saud University.

### The study instrument

A self-administered questionnaire was designed specifically for this study based on a thorough review of the related literature [15, 17, 19, 30]. The instrument was peer reviewed by three academics in the field to test its content validity. A pilot study was conducted with 20 participants to measure the reliability. Cronbach’s alpha was 88%; the detailed reliability for each of the study dimensions was as follows: caregivers’ need = 89% and caregivers’ expectations = 90%.

The questionnaire included four main parts: The first was the demographic part which asked about the caregivers’ age, gender, marital status and if having children, education, living arrangements, monthly income, and occupation. The second part asked about the care recipient’s gender, age, relation to the caregiver, living arrangements, and whether there were other individuals supporting in the caregiving process. The third part was designed using a five-level Likert scale to measure the needs of informal caregivers on three levels: *the needs on the system level* which was measured by three items: necessary equipment, alternative safe care settings when the caregiver is unavailable, and training on caring process; *the needs on the social level* which was measured by three items: free time to socialize, support by family, and support by friends; and finally, *the needs on the financial level* which was measured by four items: adequate income, housekeeping, and nurses or assistants to help in caring for care recipients.

A five-level Likert scale was used in the fourth part of the questionnaire to identify the caregivers’ expectations of formal support on three aspects: first,* the material support* which was measured by eight items: consideration of special needs in building regulations, enhancement of home healthcare services, availability of trained staff or equipped facilities to provide the care when caregivers are at work or traveling, availability of necessary equipment, and the establishment of nursing homes; second, *the information support* which was measured by four items: society awareness about disabilities, availability of consultation through phone lines and websites, and the availability of a reliable formal source of information on disability equipment; and third, *the financial support* which was measured by four items: disability adaptable transportation, flexibility at work in vacations and working hours, and fixed salary for caregivers.

The five-level Likert scales in parts 3 and 4 were coded with 5 representing the highest need/expectation and 1 representing the lowest need/expectation. The total scores were classified as follows: very low (mean = less than 1.80), low (M = 1.80–2.59), moderate (M = 2.60–3.39), high (M = 3.40–4.19), and very high (M = 4.20 and more).

### Data collection

The invitation started through an electronic survey from the 14th of November until the third of December 2015. After cleaning the data and checking for duplicate forms, a total number of 271 informal caregivers were identified as appropriate participants, representing a 90.3% response rate. The data collection process followed research ethics; it was filled out on a voluntary basis, and the participants were assured of their confidentiality and the appropriate use of their data for research purposes only. The application of the findings of this study in the development plans of formal systems to enhance the caregiving process within Saudi society was the primary benefit of this study.

### Statistical analysis

Data was analyzed using The Statistical Package for the Social Sciences (IBM SPSS Statistics for Windows, version 22.0. Armonk, NY: IBM Corp.). Demographic characteristics were described using frequencies and percentages. The needs and expectations of the caregivers were measured by calculating the means and standard deviations. Independent samples *t*-test and one-way ANOVA test were performed to test for significant differences and associations. The significance level was set at ≤ 0.05.

## Results

More than half of the study participants cared for one elderly (55%), while about 16% and 2% cared for two elderlies and more than two elderlies, respectively. On the other hand, 21% of the participants cared for one individual with disability while about 6% cared for two or more individuals. Table 1 presents a description of the participants; there were more female than male caregivers and nearly half of them were married. Most of the participants were in their twenties and thirties (70.2%), more than half had a bachelor’s degree, and almost one-half were employed.

**Table 1:** Characteristics of informal caregivers in Saudi Arabia, 2015 (*n* = 271)

Variable	Frequency	Percentage
**Gender**		
‒ Male	85	31.4
‒ Female	186	68.6
**Age**		
‒ Less than 18 years	4	1.5
‒ 18–29	100	36.9
‒ 30–39	93	34.3
‒ 40–49	48	17.7
‒ 50–59	15	5.5
‒ 60 years and more	11	4.1
**Marital status**		
‒ Single	99	36.5
‒ Married	134	49.4
‒ Widow	15	5.5
‒ Divorced\separated	23	8.5
**Having children**		
‒Yes	144	53.1
‒ No	127	46.9
**Living arrangement**		
‒ Live alone	12	4.4
‒ Live with parents	127	46.9
‒ Live with husband\wife	120	44.3
‒ Others	12	4.4
**Educational level**		
‒ Not educated	2	0.7
‒ Less than secondary	4	1.55
‒ Secondary level	56	20.7
‒ Diploma	24	8.9
‒ Bachelor	148	54.6
‒ Higher education	37	13.7
**Career**		
‒ Employee	127	46.9
‒ Student	55	20.3
‒ Employee and student	19	7.0
‒ Not an employee nor a student	70	25.8
**Monthly income**		
‒ Have no income	35	12.9
‒ Less than 3000	64	23.6
‒ Between 3000 and 8000	63	23.2
‒ Between 8000 and 12,000	67	24.7
‒ Between 12,000 and 20,000	34	12.5
‒ More than 20,000	8	3.0

As shown in Table 2, more than half of the care recipients were female and most were the parents of the caregivers. Most of them were living with their caregivers and almost half were aged 60 years or older. More than 60% of the participants (61.3%) reported that they were supported by relatives in providing care for their care recipients.

**Table 2:** Characteristics of recipients of informal care in Saudi Arabia, 2015 (*n* = 271)

Variable	Frequency	Percentage
**Gender of care recipients**		
‒ Male	115	42.4
‒ Female	156	57.6
**Age of care recipients**		
‒ 0–5	12	4.4
‒ 6–10	30	11.1
‒ 11–20	20	7.4
‒ 21–30	4	1.5
‒ 31–40	2	0.7
‒ 41–50	24	8.9
‒ 51–60	55	20.3
‒ 61 +	124	45.8
**Relationship with caregiver**		
‒ Husband\wife	4	1.5
‒ Parent\(in-law)	159	58.7
‒ Grandparent\(in-law)	36	13.3
‒ Daughter\son	40	14.8
‒ Sibling\(in-law)	25	9.2
‒ Others	7	2.6
**Living arrangement of care recipients**		
‒ Lives alone	20	7.4
‒ Lives with caregiver	177	65.3
‒ Lives with others	74	27.3
**Others help caregivers in providing care**		
‒ No	67	24.7
‒ Yes, relatives	166	61.3
‒ Yes, paid assistant	38	14.0

Table 3 presents the measurement of the caregivers’ needs throughout the caring process on three levels: the system level, the social level, and the financial level. On the system level, the *participation of formal institutions in providing necessary equipment* was the highest need reported by study participants (mean ± SD = 4.47, 0.802). On the social level, *the caregivers’ need for free time to engage more in social activities* was the highest need reported (M ± SD = 4.30, 0.912), whereas on the financial level, the highest need reported was *an appropriate income to support caregivers in their daily living obligations* (M ± SD = 4.20, 1.005). However, the caregivers’ need on the system level was the highest among the measured needs and the overall needs of the participants were reported to be on a high level.

**Table 3:** Needs of informal caregivers in Saudi Arabia, 2015 (*n* = 271)

**Aspects**^b^	**Mean**^a^	**SD**
**Needs on the system level**		
The involvement of hospitals and public agencies in providing the necessary equipment for the person you are taking care of (to raise independence and support)	4.47	0.802
Safe alternatives for the person you are taking care of provided by official agencies in case of being busy	4.23	1.021
Training courses that enable you to deal with your care recipient in a better way	4.06	1.015
Overall needs on the system level	4.25	0.787
**Needs on the social level**		
Free time for participating in social activities	4.30	0.912
A member of the family taking care of the person that you are taking care of in case of being busy	4.11	0.996
One of your close friends that can take care of the person you are taking care of in case of being busy	3.22	1.196
Overall needs on the social level	3.87	0.758
**Needs on the financial level**		
Suitable income helps you cover the main needs of daily life for you and the person you are taking care of	4.20	1.005
A servant undertakes your familial responsibilities that are difficult for you to handle, as you are busy with your care recipient	4.11	1.138
Professional assistant “nurse” who is brought in as a nurse to help you take care of the person you are taking care of	3.22	1.196
Non-professional assistant to help you in taking care of the person you are taking care of	3.55	1.134
Overall needs on the financial level	3.96	0.810
**Overall needs of informal caregivers**	**4.02**	**0.658**

^a^On the five-level Likert scale, 5 represents the highest expectation and 1 represents the lowest expectation

^b^Items listed based on the degree of expectations starting with the greatest

Table 4 shows the caregivers’ expectations of support mechanisms from the formal systems on three aspects: material, information, and financial. *Having public facilities built in a way suitable for dependent individuals* was reported as the highest expected material support. *Increasing public awareness regarding the needs of individuals with any sort of dependency* was reported as the highest expected information support. Lastly, *the availability of disability-friendly transportation* was reported as the highest expected financial support. Overall, the participants expected a great deal of formal support whereas their greatest expectations were on the information support.

**Table 4:** Informal caregivers’ expectations of the formal system in Saudi Arabia, 2015 (*n* = 271)

**Aspects**^b^	**Mean**^a^	**SD**
**Expectations of material support**		
Suitability of public facilities for care recipients	4.70	0.663
Expansion of home healthcare services	4.65	0.687
Trained individuals to substitute caregivers while at work	4.51	0.843
Availability of necessary equipment for purchasing out of pocket	4.34	0.920
Well-established facilities for care recipients—temporary residency	4.27	0.896
Trained individuals to substitute caregivers while on travel	4.21	1.008
Well-established facilities for care recipients—permanent residency	4.13	0.977
Well-established facilities for care recipients—residency while caregiver at work (respite care)	4.12	1.037
Overall expectations of material support	**4.36**	**0.632**
**Expectations of information support**		
Society awareness about disability	4.75	0.593
Reliable information source (phone consultation)	4.56	0.845
Reliable information source (online consultation)	4.55	0.782
Reliable equipment information source	4.46	0.889
Overall expectations of information support	**4.58**	**0.649**
**Expectations of financial support**		
Disability-friendly transportation	4.67	0.731
Flexibility in job vacation schedules	4.52	0.816
Flexibility in job working hours	4.50	0.816
Fixed income for informal caregivers	4.26	1.023
Overall expectations of financial support	**4.49**	**0.647**
**Overall expectations of the formal system**	**4.45**	**0.572**

^a^On the five-level Likert scale, 5 represents the highest expectation and 1 represents the lowest expectation

^b^Items listed based on the degree of expectations starting with the greatest

Independent samples’ *t*-tests were conducted to examine whether there were significant differences between caring for an elderly and caring for an individual with disability in relation to caregivers’ needs and expectations. Caring for either type of dependency did not differ in relation to the needs of the caregivers whereas the expectations of the caregivers differed significantly; caregivers who were caring for individuals with disabilities expected more of the formal system (mean ± SD = 4.60, 0.41) than those who were caring for elderly individuals (mean ± SD = 4.40, 0.61; *t* (269) =  − 2.595, *p* = 0.006). Specifically, they differed in two aspects of support expectations: caregivers of individuals with disability expected more information support (mean ± SD = 4.78, 0.42) than caregivers of elderly individuals (mean ± SD 4.51, 0.70; *t* (269) =  − 3.071, *p* ≤ 001). At the same time, caregivers of individuals with disability expected more financial support (mean ± SD = 4.66, 0.53) than caregivers of elderly individuals (mean ± SD = 4.43, 0.68; *t* (269) =  − 2.634, *p* = 0.025).

## Discussion

This study measured the perception of the participants towards their needs and expectations that have been shaped by their experience of caregiving. *First*, the needs of the informal caregivers were identified on three levels through ten items, most of which supported the previous findings. The great need of the current participants for the provision of necessary equipment for their dependent individuals concurred with the findings reported by caregivers of terminally ill patients [23]. A previous study [16] that emphasized the need for nursing homes when the family is no longer able to provide care was supported in this study by the participants expressing their need for safe alternative caring settings when needed. In support of previous findings [12, 22, 27, 28, 31], the participants of this study also stressed their needs for training courses to enhance the caring process.

In accordance with previous findings [13, 14], the current participants reported a greater need for free time to enhance their social activities as well as the need for family help in the caring process. Studies investigating informal caregiving in Malaysia, a society that resembles the Saudi society, reported urgent needs for social support [30, 32]. Other societies have been reported to be experiencing some deficiencies in social support for informal caregivers such as the Dutch, the British, and the Indian [15, 22, 31]. Even though most of the developed systems support their informal caregivers more compared to other systems, apparently, the need for social support in those developed societies is still an issue and cannot be fully replaced.

The current study revealed a greater need for system support than for social support though the latter was perceived by the participants as a high need. Less than two-thirds of the participants reported receiving assistance from family members throughout the caring process to match up with the findings of a previous study which concluded that in Saudi Arabia, informal caregiving requires greater family support and formal support among other unrecognized needs [29]. Up until two decades ago, the family structure was thought to be very strong; family ties and closeness were not limited to the parents and the children, they extended to include relatives of different degrees. The strong interpersonal bonds between the patient and his/her family and his/her community along with the Islamic view of the wellbeing of a person along with the emphasis on faith in Allah had helped alleviate some of the impacts of pain and sorrow [28]. Apparently, Saudi society has gone since then through the same transitional phase that other societies have experienced. Accordingly, relying heavily on the earlier image of social support would not be in favor of the caregivers.

Although most of the participants had moderate to low monthly income, they did not emphasize the financial needs that much; their needs for equipment for their care recipients, alternative caring settings, and free time to socialize were greater than the need for proper income. In 2014, a study emphasized that caregiving may negatively affect caregivers’ job performance and reduce their chances of promotion too; thus, many become in real need for government support [30]. That finding was pointed out clearly in the present study; given that more than half of the participants were employed, the need on the system level was the highest compared to the need on both financial and social levels.

*Second*, the perceived expectations that have been shaped by the experience of caregiving were identified in relation to three aspects through 16 items, some of which supported previous findings. The participating caregivers expected the formal system to provide long-term care within well-equipped facilities, which is consistent with previous findings [23, 27, 28], as well as within recipients’ home. Even though the Saudi health system is comprehensive and free for all citizens [29], its deficiencies in long-term care [27, 28, 31] would make the process of informal caregiving more problematic. Eventually, the caregivers emphasized enhancing the scope of long-term care, a great mechanism in which formal caregivers complement the role of informal caregivers.

In support of the literature [13, 16, 22, 23, 26], the participants expected the formal system to provide reliable and accurate sources of information on the caring process. Among the 16 items measuring caregivers’ expectations, the greatest expressed ones were regarding mobility within the community. Although the participants reported great expectations for each source of information, their emphasis was more on *enhancing the awareness of the society of dependency and its needs*. On the material aspects, their greatest expectation was the *suitability of public buildings to individuals with disability*. On the financial aspects, their greatest expectation was *the availability of equipped transportation for individuals with disability.* Evidently, informal caregivers have been facing difficulties in merging their care recipients within the community which requires great attention. A study conducted in Oman, a neighbor country to Saudi Arabia, had noticed that many caregivers cannot take their autistic children to public areas as their behavior is likely to attract the attention of others [20]. The society is totally responsible for merging all its members within it; it should not be left to the families to deal with it as a family issue nor should it be ignored. Clearly, the strategies adopted to merge individuals with disability within Saudi society need reevaluation; the society must be able to absorb those individuals with their special needs in a more mature way.

Across European countries, direct or indirect financial support has been developed to compensate caregivers [25]. Pertaining to this point, Saudi caregivers, through the responses of the current participants, reported great expectations of a fixed salary as a support mechanism from the formal authority. Finally, the participating caregivers expected flexibility in work regulations regarding working hours and vacation schedules to enhance the caregiving process which corresponded to previous findings [20, 30].

Ultimately, most of the study participants were female and in their reproductive age, which would generate more challenges due to the higher burden of responsibilities, knowing that a quarter of the current caregivers were caring for two or more dependent individuals. Additionally, except for a few participants, the monthly income was reported as being moderate to low.

### Limitations of the study

Despite the detected challenges within the current data, it should be considered with some caution; more than a third of the care recipients were not living with the caregivers, whereas two-thirds of the caregivers had been assisted by family members in the caregiving process. The participants might be informal caregivers but not all of them were the primary caregivers. Eventually, information about Saudi informal caregivers and their prevalence is quite limited as well as the fact that they are a target population that is hard to access.

## Conclusion

Saudi caregivers are experiencing various needs within their informal social role, and they are expecting, especially those caring for individuals with disability, greater support from the society. They deserve recognition and consideration in developmental research. The international literature can be utilized to supplement the shortages in Saudi literature given that most of the emphasized needs and expectations among the current participants were in correspondence with those experienced by informal caregivers in other societies. A longitudinal study would be an appropriate method of measuring and explaining the experience of caregiving, with a special emphasis on the extent of the caring process and clarification of the roles provided within that process. The authors recommend considering long-term care alternatives, along with well-equipped facilities, and support mechanisms when planning healthcare services. Organizing the care services, along with initiating a database, and work/family regulations would ensure better care with more manageable efforts. Additionally, disability inclusion in all levels of the health system is highly recommended, specifically in primary healthcare.

## Data Availability

The dataset generated and analyzed during the current study is available from the first author on reasonable request.

## Abbreviations

ADLs: Activities of daily living
IADLs: Instrumental activities of daily living
WHO: World Health Organization
SPSS: Statistical Package for the Social Sciences
ANOVA: Analysis of variance
SD: Standard deviation
